# Definitive salvage radiation therapy and chemoradiation therapy for lymph node oligo-recurrence of esophageal cancer: a Japanese multi-institutional study of 237 patients

**DOI:** 10.1186/s13014-017-0780-5

**Published:** 2017-02-20

**Authors:** Hideomi Yamashita, Keiichi Jingu, Yuzuru Niibe, Kuniaki Katsui, Toshihiko Matsumoto, Tomohiro Nishina, Atsuro Terahara

**Affiliations:** 1 Department of Radiology, the University of Tokyo, 7-3-1, Hongo, Bunkyo-ku, Tokyo, 113-8655 Japan; 2 Department of Radiation Oncology, Tohoku University Graduate School of Medicine, 1-1, Seiryou-cho, Aoba-ku, Sendai, 980-8575 Japan; 3 Department of Radiology, Toho University Omori Medical Center, 6-11-1, Omori-nishi, Ota-ku, Tokyo, 143-8541 Japan; 4 Department of Proton Beam Therapy, Okayama University Graduate School of Medicine, Dentistry and Pharmaceutical Sciences, 2-5-1, Shikata-cho, Kita-ku, Okayama 700-8558 Japan; 5 Department of Gastrointestinal Medicine, Shikoku Cancer Center, Kou 160, Umemoto-cho, Matsuyama, Ehime 791-0280 Japan; 6 Department of Internal Medicine, Himeji Red Cross Hospital, 1-12-1, Shimoteno, Himeji, Hyogo 670-8540 Japan

**Keywords:** Esophageal cancer, Oligo-recurrence, Oligometastases, Salvage chemoradiation therapy, Salvage radiation therapy

## Abstract

**Background:**

This study evaluated the treatment results of lymph node (LN) oligo-recurrence in esophageal cancer patients treated with salvage radiotherapy (RT) in a multi-institutional retrospective study.

**Methods:**

Eligibility criteria for this retrospective analysis were: the primary lesion of esophageal cancer was controlled; from one to five LN recurrences; total RT dose ≥45 Gy to exclude palliative RT; without recurrence other than LN; and salvage RT for LN recurrence was given between January 2000 and April 2015. The median follow-up time for the 93 living patients was 29.6 months.

**Results:**

Two hundred thirty-seven patients were matched in five hospitals. The 3-year overall survival (OS) was 37%, local control was 45%, progression-free survival was 24%, and esophageal cancer-specific survival was 42%. On univariate analysis for OS, combined chemotherapy (*p* = 0.000055), disease-free interval (DFI) ≥12 months (*p* = 0.0013), LN max diameter ≤22 mm (*p* = 0.0052), and Karnofsky performance status ≥80% (*p* = 0.030) were associated with a significantly better prognosis. On multivariate analysis, significant differences were seen for combined chemotherapy (*p* = 0.000018), DFI (*p* = 0.0027), and LN max diameter (*p* = 0.018).

**Conclusions:**

LN oligo-recurrence following treatment for esophageal cancer was not a terminal-stage event. Moreover, cure may be possible by chemoradiation therapy with a long DFI (≥12 months) and small size (≤22 mm).

## Background

Lymph node (LN) recurrence from esophageal cancer after surgery is one of the main types of treatment failure [1–3]. According to several reports, 42.5–52.4% of operated patients develop recurrence, and these patients’ prognosis remains poor [4–8]. The median survival time (MST) of all postoperative recurrent esophageal cancers including loco-regional, distant, and combined recurrence has been shown to be 6.0–8.2 months [1, 5]. Although some analyses indicated that treatment of locoregional recurrence (LR) prolonged survival regardless of the treatment type, the outcome of patients treated with chemotherapy (CTx) alone was significantly worse than for patients treated with other intensive therapies [9]. Therefore, CTx alone is usually reserved for patients with distant metastases.

On the other hand, radiotherapy (RT), chemoradiation therapy (CRT), or lymphadenectomy have been used in treating LR. These treatments have had a beneficial symptomatic effect for a significant proportion of these patients, and it is possible to obtain long-term survival in some patients [9–12]. Cancer patients with ≤ 5 metastatic or recurrent lesions with controlled primary lesions can be considered as having "oligo-recurrence". The concept of oligo-recurrence was proposed and defined by Niibe *et al.* in 2006 [13–15]. Local therapy was occasionally added to these recurrent sites with or without systemic therapy. However, the outcome of salvage RT and prognostic factors for LN oligo-recurrence of esophageal cancer have not been studied extensively. In some institutions, local therapy was given to patients without controlled primary lesions, but this was based on the assumption of radical resection for primary lesions after local therapy for recurrence.

For LN oligo-recurrence after primary definitive CRT, salvage surgery is first considered, and, when surgery is not indicated, salvage CRT or stereotactic radiotherapy is performed. Some researchers [16, 17] have also advocated that, for clinically isolated locoregional recurrence or clinically solitary solid organ metastasis patients, surgical therapy with or without systemic therapy should be considered first after diagnosis of recurrence, although it may not be a universal approach. Additionally, they have argued that, when surgery was impossible or contraindicated, the combination of CRT appeared to be superior to chemotherapy alone or radiotherapy alone [18, 19]. In some institutions, for inoperable LN recurrence after radical surgery or definitive CRT, curative therapy is abandoned, and palliative CTx or best supportive care is selected.

The purpose of this retrospective study was to assess the efficacy of salvage RT or CRT for inoperable LN recurrence after primary curative therapy for esophageal cancer.

## Methods

### Subjects

The eligibility criteria for this retrospective analysis were as follows: a) the primary lesion of esophageal cancer was controlled; b) from 1–5 LN recurrences; c) total RT dose of ≥45 Gy in order to exclude palliative RT; d) without recurrence other than LN; and e) salvage RT or CRT for LN recurrence was given between January 2000 and April 2015. The eligibility criteria did not include with or without combined CTx, the kind of initial curative therapy, the disease-free interval (DFI), recurrent LN location, LN max diameter, age, Karnofsky performance status (KPS), and the histopathological type of primary tumor.

The DFI was defined as the interval between initial therapy for the primary lesion and the date of identification of LN recurrence. This was the endoscopic submucosal dissection date, the operation date independent of perioperative CTx, or the starting date of initial CRT.

The reason why the 11 patients with stage IV disease received curative treatment (surgery in eight patients and CRT in three) was that these patients had only supraclavicular, para-aortic, or hilar LN metastasis, and cure was considered possible. These stages were classified based on the UICC/AJCC TNM system version 7.

### Statistical analysis

Survival curves were prepared using the Kaplan-Meier method, and the *p* value on univariate analysis for overall survival (OS) was calculated by the log-rank test. The 95% confidence interval (CI) was calculated using Greenwood’s formula. The significance level was set at 5%. The events were defined as any death for OS, local recurrence within the radiation field for local control, any death and any relapse for relapse-free survival, and death from esophageal cancer for esophageal cancer-specific survival. Multivariate analysis for OS was performed with a Cox proportional hazards model, and the variables were selected by the stepwise method using the Bayesian Information Criterion (BIC). The Bonferroni correction was used for multiple comparisons; in other words, the significance level was set as 5% divided by the number of variables in the multivariate analysis.

## Results

### Patient and tumor characteristics

A total of 237 patients who matched the study definition of oligo-recurrence were treated by CRT or RT alone in five Japanese hospitals. The median age was 66 years (range, 36–87 years). The male-to-female ratio was 207:30. The ratio of KPS ≥90% to <90% was 154:83. Primary histopathology was squamous cell carcinoma (SCC) in 231 patients, adenocarcinoma or adeno-squamous cell carcinoma in three patients, and others in three patients. Clinical stages I, II, III, and IV at the initial curative therapy were seen in 34, 89, 103, and 11 patients, respectively. The primary tumor location was cervical in 10 patients (whose primary therapy was surgery in 9 patients and CRT in one patient), upper thoracic in 22 patients, middle thoracic in 140 patients, and lower thoracic plus gastric-esophageal junction in 65 patients. The primary therapy was endoscopic submucosal dissection in five patients, radical surgery in 219 patients (including preoperative chemoradiation in three patients, preoperative CTx in two patients, postoperative CTx in one patient), and definitive CRT in 13 patients. The median DFI was 11.9 months (range, 1.1–149.1 months). The number of LN recurrences was one in 161 patients (68%), two in 31 patients, three in 29 patients, four in seven patients, and five in five patients. The median maximum LN diameter (MLD), which was defined as the greatest transverse diameter in the axial plane, was 22 mm (range, 5–106 mm). The location of recurrent LN metastasis was loco-regional only in 144 patients (60.8%) and distant regions only, such as neck, supraclavicular, abdominal para-aortic, or hilar LNs, in 66 patients (27.8%); 27 patients (11.4%) had both loco-regional and distant region involvement. The median total radiation dose was 60 Gy (range, 45–70 Gy). Median overall treatment time was 43 days (range, 11–57 days). Radiation therapy was involved-field in 170 patients, extended-field including prophylactic irradiation in 62 patients, and stereotactic body radiotherapy of 50 Gy in 10 fractions in five patients. Systemic CTx was administered concurrently to 199 patients (84%), but two patients received it sequentially following RT. The regimen included an FP regimen (5-fluorouracil and cisplatin) in 65 patients, nedaplatin plus 5-fluorouracil in 102 patients, S1 alone in 17 patients, docetaxel alone in nine patients, a DCF regimen (docetaxel, cisplatin, and 5-fluorouracil) in two patients, and others in four patients.

### First failure site

The first failure site after this definitive salvage RT was lung in 38 patients, liver in 19 patients, bone in 10 patients, other distant organs in 11 patients, distant LN in 47 patients, and locoregional LN in 61 patients (31 patients within field and 30 patients outside field). When taken together, in-field recurrence of salvage RT occurred in 31 patients (16.7%), and out-of-field recurrence was seen in 155 patients (83.3%).

### Survival

The median follow-up time for the 93 living patients was 29.6 months (range, 1.9–154.0 months). The overall follow-up was 15.0 months (range, 0.2–154 months), and 12 patients were lost to follow-up. The 3-year OS was 36.7% (95% CI: 29.8–43.6%), local control was 45.1% (95% CI: 37.3–52.6%), relapse-free survival was 24.1% (95% CI: 18.6–30.1%), and esophageal cancer-specific survival was 41.5% (95% CI: 34.1–48.7%). The MST was 21.6 months (95% CI: 18.0–28.5 months).

### Univariate analysis of OS (Table 1)

**Table 1:** Univariate analysis of overall survival

Factors	No.	MST	95% CI	*p* value
Age, y				
> 66	109	21.5	15.3–32.6	0.76
≤ 66	128	21.6	14.6–29.1	
Serum SCC antibody				
> 2 ng/mL	65	23.0	10.0–29.0	0.42
≤ 2 ng/mL	172	21.6	18.0–30.6	
Combined CTX				
Without	38	10.8	6.3–21.5	0.000055
With	199	26.0	18.5–32.8	
DFI				
≤ 12 mo	119	14.5	12.8–18.8	0.0013
> 12 months	118	30.6	23.2–42.0	
DFI				
≤ 24 months	183	18.0	14.2–23.2	0.0041
> 24 months	54	41.0	26.0–116.0	
KPS				
< 90%	83	14.6	12.3–28.0	0.11
≥ 90%	154	26.0	18.0–32.8	
No. of LN metastasis				
Mono	165	26.0	17.8–33.0	0.44
Multiple	72	18.8	14.6–28.5	
LN max diameter				
> 22 mm	109	15.7	13.5–23.5	0.0052
≤ 22 mm	128	29.1	19.8–41.1	
LN location				
Distant	90	20.2	14.2–27.0	0.41
Locoregional	147	26.4	18.0–34.3	
Histopathology				
SCC	231	23.0	18.0–28.5	0.55
Others	6	15.0	12.0-NA	

*Abbreviation*: *No*. number, *MST* median survival time, *CI* confidence interval, *SCC* squamous cell carcinoma, *CTX* chemotherapy, *DFI* disease-free interval, *KPS* Karnofsky performance status, *LN* lymph node

**Fig. 1:**
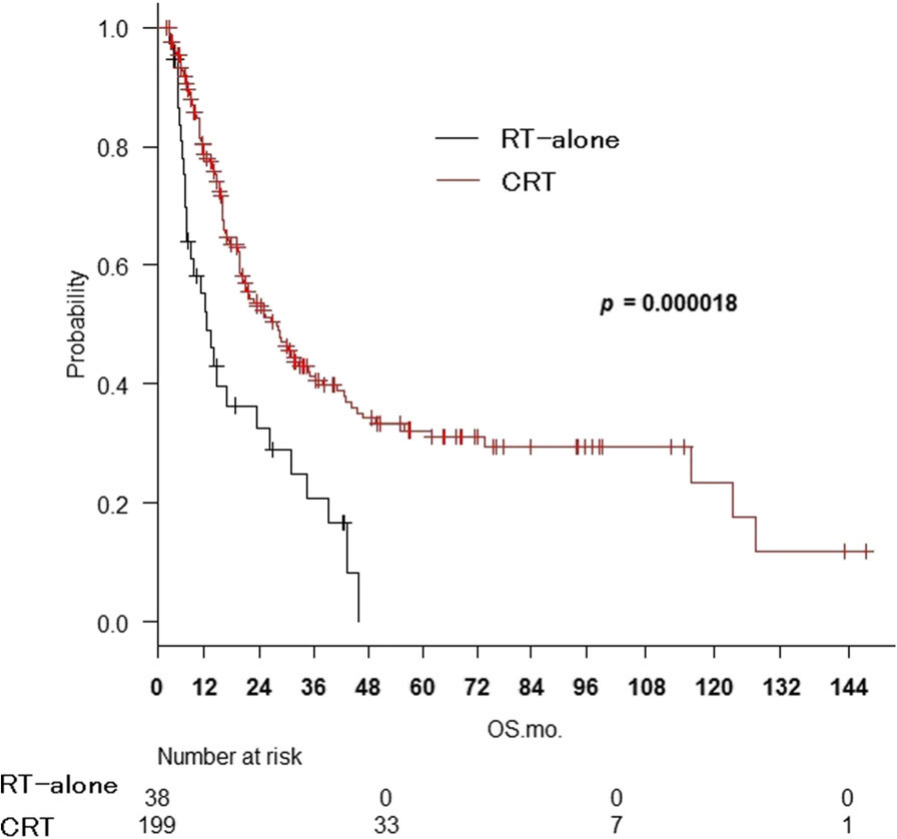
Overall survival curves for CRT and RT alone

**Fig. 2:**
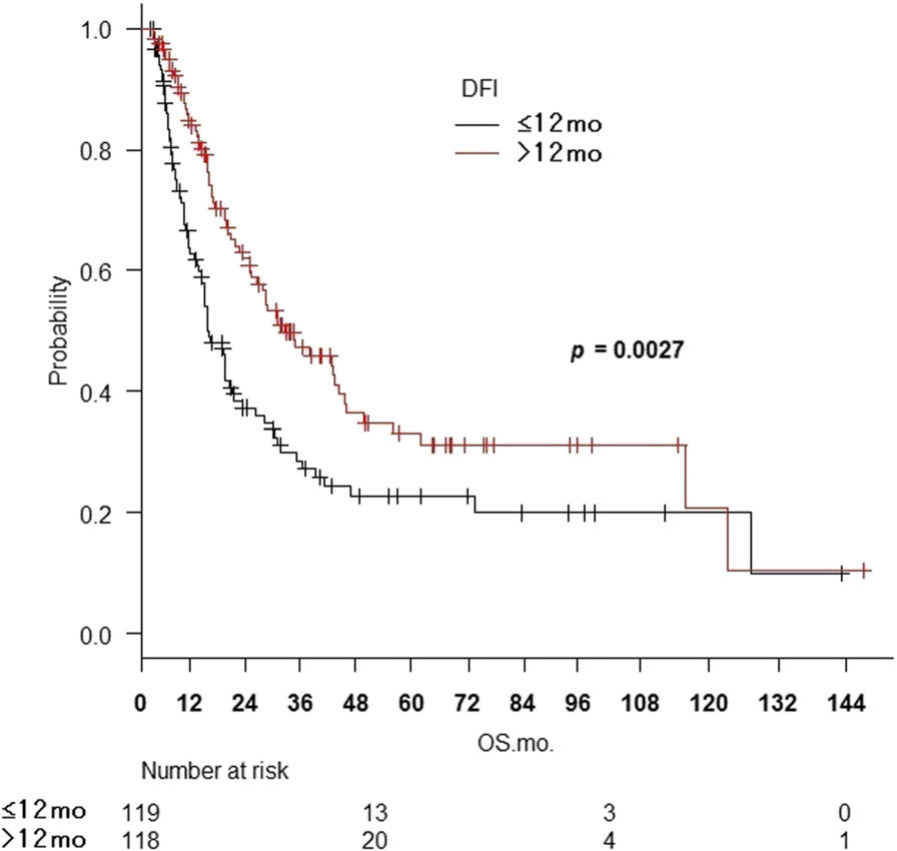
Overall survival curves for disease-free interval < 12 months and ≥ 12 months

**Fig. 3:**
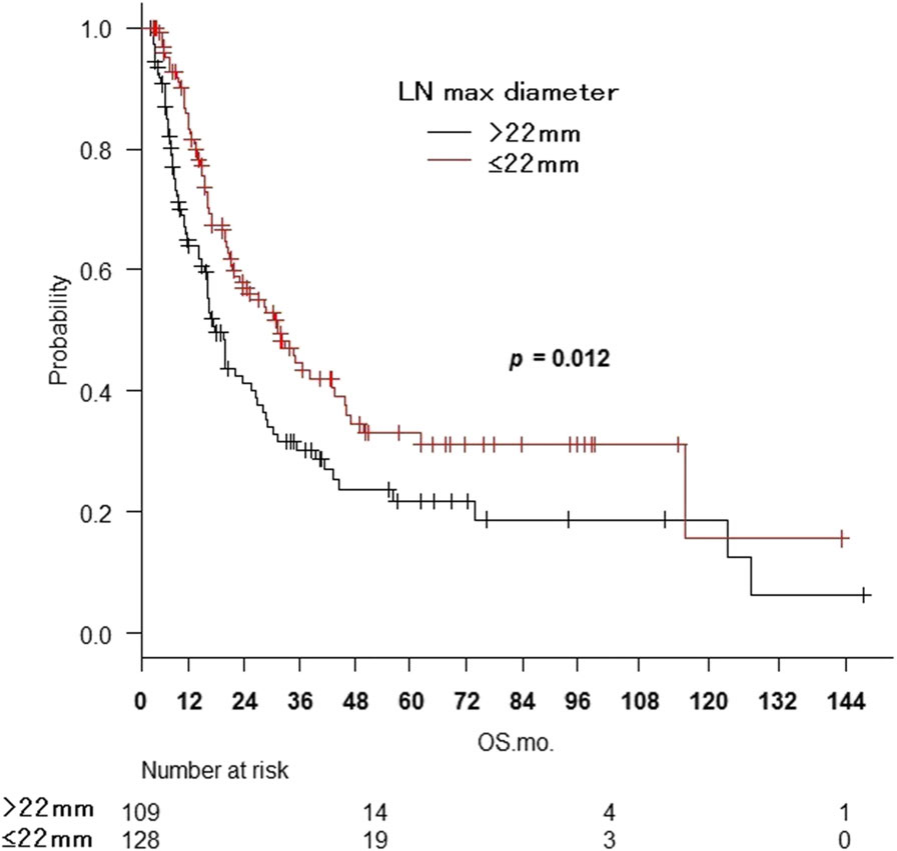
Overall survival curves for recurrent lymph node maximum diameter > 22 mm and ≤ 22 mm

The 3-year OS was 39.7% (95% CI: 32.1–47.3%) with CRT and 20.8% (95% CI: 8.3–37.0%) with RT alone (*p* = 0.000055, log-rank test) (Fig. 1). The 3-year OS was 45.9% (95% CI: 35.6–55.6%) for DFI ≥12 months and 27.3% (95% CI: 18.6–36.6%) for DFI <12 months (*p* = 0.0013) (Fig. 2). The 3-year OS was 30.2% (95% CI: 20.1–39.9%) for MLD >22 mm and 42.1% (95% CI: 32.3–51.6%) for MLD ≤ 22 mm (*p* = 0.0052) (Fig. 3). The KPS 80–100 group achieved a 3-year OS of 43.1%, compared to 11.9% for KPS ≤70 (*p* = 0.030). Age >66 vs. ≥66 years (*p* = 0.76), KPS >90% vs. ≤90% (*p* = 0.11), number of LN recurrences mono vs. multiple (*p* = 0.44), LN location locoregional vs. distant (*p* = 0.41), serum SCC antibody value >2 mg/mL vs. ≤2 mg/mL (*p* = 0.42), and histopathological type SCC vs. others (*p* = 0.55) were not significant prognostic factors.

### Multivariate analysis of OS

On multivariate analysis by the BIC stepwise method (Table 2), multiple factors such as age, KPS, number of LNs, LN location, histopathological type, serum SCC antibody value, CTx, DFI, and LN max diameter were included. CTx (HR = 0.40 and *p* = 0.000018), DFI (HR = 0.60 and *p* = 0.0027), and LN max diameter (HR = 0.65 and *p* = 0.012) were significant.

**Table 2:** Multivariate analysis of overall survival by the stepwise method

Factors	HR	Lower	Upper	*p* value
		95% CI	95% CI	
CTX				
Without/with	0.40	0.27	0.84	0.000018
DFI				
≤/> 12 months	0.60	0.43	0.84	0.0027
LN max diameter				
>/≤ 22 mm	0.65	0.43	0.84	0.012

*Abbreviation: HR* hazard ratio, *CI* confidence interval, *CTX* chemotherapy, *DFI* disease-free interval, *LN* lymph node

The number of patients with all of (a) treated with CRT, (b) DFI of not less than 12 months, and (c) LN max diameter of not more than 22 mm was 56 (24%). The 2-y OS and 3-y OS of these patients were 64.9% (95% CI: 50.1–76.3%) and 55.2% (95% CI: 40.0–68.0%), respectively, and the MST was 43.4 months (95% CI: 26.5-NA months).

### Toxicity

Eleven patients (4.6%) experienced acute or late non-hematologic adverse events of grade 3 or greater according to the Common Terminology Criteria for Adverse Events (CTCAE) v4.0, including: grade 4 cardiac tamponade at 4.6 months after the completion of RT; grade 5 drug-induced interstitial pneumonia at 0.3 months; grade 4 hyperglycemia during treatment; grade 5 pleural effusion at 13.5 months; grade 3 anastomotic stenosis at 1.4 months; grade 5 mediastinal-bronchial fistula at 2.6 months; grade 5 esophageal bleeding at 3.3 months; grade 4 esophagobronchial fistula during treatment; grade 5 gastric ulcer bleeding at 0.2 months; grade 4 fistula of a gastric tube at 15.4 months; and grade 4 gastric tube to bronchial fistula at 1.0 months.

## Discussion

According to previous studies of RT or CRT for patients with postoperative LR of esophageal cancer, the median MST of 13 studies was 13.6 months (range, 7–24.3 months), and the median 2-y OS was 25% (range, 10.5–51%) [9–11, 20–25]. Among these studies, Jingu et al. [12] reported the long-term results of CRT for postoperative LR in their prospective phase II study. A total of 30 patients were treated for postoperative LR with RT (60 Gy in 30 fractions) combined with CTx consisting of two cycles of nedaplatin (70 mg/m^2^/day) and 5-fluorouracil (500 mg/m^2^/day, for 5 days). With a median observation period of 72 months, the 3-y OS was 38.4%, with an MST of 21.0 months. Three-year relapse-free survival was 29.3%, and the 3-y irradiated-field control rate was 71.5%. Although the present results should be interpreted with caution because of the short observation period, the MST of 21.6 months and the 3-y OS of 36.7% can be looked upon as favorable and encouraging.

Several prognostic factors have been reported. For example, high RT dose, younger age, non-anastomotic recurrence, good performance status, single LN recurrence, and single recurrent region predicted better outcomes [9–11, 20, 21, 24–26]. The optimal RT dose for LR has not been established. Some studies demonstrated that a high RT dose was a better prognostic factor [10, 11, 25]. Zhang et al. [11] reported that an RT dose of more than 60 Gy showed a trend to improving OS. In the present study, univariate analyses showed that the significant prognostic factors for OS were CRT, DFI ≥12 months, MLD ≤22 mm, and KPS ≥80%.

In the study of Jingu *et al.* [12, 24], patients who had LN metastases in multiple regions, such as mediastinal and supraclavicular or mediastinal and abdominal LNs, or metastases to many LNs in one region were irradiated by a T-shaped field (including the bilateral supraclavicular, mediastinal, and abdominal regions). Especially without combined chemotherapy, prophylactic irradiation may be needed at some level in view of the involvement of the lymphatic system for such patients with LN recurrence. In the present study, there was no difference in OS between elective lymph node irradiation and involved-field radiotherapy.

The present study has several limitations associated with its retrospective design. This study could not demonstrate a survival benefit of salvage RT or CRT compared to other treatment modalities such as CTx-alone. According to the previous reports of life-prolonging chemotherapy alone for esophageal M1 patients, the median survival time was 10.0–15.5 months [16, 17, 27, 28]. In the present study, the MST was 21.6 months, and the lower value of the 95%CI was 18.0 months. Based on this result, at least the combination of CTx with RT for oligo-recurrence in the LNs from esophageal cancer is strongly recommended, although the inclusion criteria of this study were quite different from esophageal M1 chemotherapy trials in which patients with poorer risk were also included. There were some selection biases: the selection of primary therapy and/or salvage therapy was different among these five institutions; whether curative salvage CRT was performed for recurrences without controlling the primary lesion; and the non-uniformity of the combined CTx regimen, RT dose, indication of stereotactic body radiotherapy, and follow-up method by institutions.

Depypere et al. [19] and Nakamura et al. [9] advocated that the combination of CRT appeared to be superior to CTx alone or RT alone for recurrent disease after esophagectomy, and the addition of CTx seems to play a crucial role in this disease state. In this study, RT alone and stereotactic radiotherapy alone were performed only for patients to whom CTx could not be delivered due to poor PS, high age, or impaired renal function.

In the present study, 60 Gy was given to 157 patients (78.9%) and 50/50.4 Gy was given to 21 patients (10.6%) of 199 patients treated with CRT. In the primary treatment, 50–50.4 Gy of RT is standard for patients treated with definitive CRT [29], although higher doses may be appropriate for tumors of the cervical esophagus, especially when surgery is not planned. However, in Japan, many hospitals have still adopted 60 Gy in place of 50–50.4 Gy, and 60 Gy represented a large percentage in the present study as well.

According to the above-cited study by Nakamura et al. [9], the 3-y cumulative OS after lymphadenectomy for 19 patients with LN recurrence of esophageal carcinoma after curative resection was 50.7%. In the present study, the 3-y OS was 36.7% (95% CI: 29.8–43.6%), and this value seemed to be slightly inferior to Nakamura’s result, although the treatment-related toxicity could not be compared.

## Conclusions

Oligo-recurrence involving LNs following treatment for esophageal cancer does not appear to be a terminal-stage event, unlike other distant metastases. Moreover, cure may be possible by chemoradiation therapy with a long DFI (≥12 months) and a small size (≤22 mm).

## Abbreviations

BIC: Bayesian information criterion
CI: Confidence interval
CRT: Chemoradiotherapy
CTCAE: Common terminology criteria for adverse events
CTx: Chemotherapy
DCF: Docetaxel, cisplatin, and 5-fluorouracil
DFI: Disease-free interval
KPS: Karnofsky performance status
LN: Lymph node
LR: Locoregional recurrence
MLD: Median maximum LN diameter
MST: Median survival time
OS: Overall survival
RT: Radiotherapy
SCC: Squamous cell carcinoma.
